# Magnetic Hygiene

**Published:** 1888-11

**Authors:** S. Helen Clarke


### MAGNETIC HYGIENE.

PART I.

Among the various forces playing an important part in the economy of nature, none probably is more directly related to the preservation and recuperation of health than magnetism.

Intimately connected or identified with life in all its forms, from the apparently unconscious, unintelligent rock, to the complex organism of the highest type of intelligent and conscious being, this subtle something, be it a fluid, a substance, or a mode of motion, exerts its powerful and vitalizing influence over the whole of creation.

It is not our purpose to follow it in its stupendous effects upon the elements constituting our planet; to travel along the subterraneous arteries with which it furrows the solidified crust of our globe, to sail upon the powerful currents with which it girts our oceans, or listen to its mighty voice in the roar of the hurricane and the howling of the tempest. We shall be satisfied with the more humble task to partially examine its relations to the human organism and briefly indicate how to practically apply the knowledge of these relations to benefit our daily lives.

We will start with the premises, that the earth is a magnet, and that man a microcosm, is a magnet, also. The property of all magnets is to attract substances or fluids in sympathy or affinity with them, and to load with or impart their magnetic properties to these. For example, iron and steel are strongly attracted to the magnet and soon become magnetized by contact with it. In the same manner our earth transmits its magnetism to all organisms which have birth and growth upon its surface, receiving in turn from the sun a constant supply of vitalizing force and power.

It is, therefore upon these conditions of attraction, transmission and absorption as human magnets, that we must base our system of magnetic hygiene.

To facilitate our labor we shall divide magnetic force into three classes: Mineral magnetism, which is the crude power manifesting itself through all nature; animal magnetism, which is of a higher degree, associated with organisms endowed with more or less intellect and will; and spiritual magnetism, which is solely an attribute of the human spirit or soul.

Man's organism and life are so deeply influenced by this power in all its aspects, that no friction of the human body, no movements of its muscles are possible without its aid.

No thought can be conveyed from the conscious and spiritual part of man to the brain and from there to the muscles, which translate that thought into objectiveness by their labor, without magnetic force becoming the vehicle of the thought and the transmitter of the will.

Without an adequate supply of magnetic force the nervous system, a most wonderfully delicate and sensitive mechanism, soon loses its elasticity and strength, and not only are the functions of circulation and nutrition quickly impaired as a result, but the intellectual faculties also become weakened in their manifestation and disturbed in their equilibrium, until the general health of the body and the power of the mind give way separately or together.

The province of hygiene is to avert such a catastrophe, and as long as we can trace the incipiency of many diseased conditions to exhausted magnetic force or vitality, we should apply hygienic action to prevent all unnecessary loss of that power, as well as to promote a supply of it adequate to a healthful condition of body and vigorous state of mind.

S. Helen Clarke.

